# Global Routine Vaccination Coverage, 2013

**Published:** 2014-11-21

**Authors:** Jennifer B. Harris, Marta Gacic-Dobo, Rudolf Eggers, David W. Brown, Samir V. Sodha

**Affiliations:** 1 Global Immunization Division, Center for Global Health, CDC; 2 Epidemic Intelligence Service, CDC; 3 Department of Immunization, Vaccines and Biologicals, World Health Organization; 4 Division of Data, Research and Policy, United Nations Children’s Fund

In 1974, the World Health Organization (WHO) established the Expanded Program on Immunization to ensure that all children have access to routinely recommended vaccines (1). Since then, global coverage with the four core vaccines (Bacille Calmette-Guérin vaccine [for protection against tuberculosis], diphtheria-tetanus-pertussis vaccine [DTP], polio vaccine, and measles vaccine) has increased from <5% to ≥84%, and additional vaccines have been added to the recommended schedule. Coverage with the third dose of DTP vaccine (DTP3) by age 12 months is a key indicator of immunization program performance. Estimated global DTP3 coverage has remained at 83%–84% since 2009, with estimated 2013 coverage at 84%. Global coverage estimates for the second routine dose of measles-containing vaccine (MCV2) are reported for the first time in 2013; global coverage was 35% by the end of the second year of life and 53% when including older age groups. Improvements in equity of access and use of immunization services will help ensure that all children are protected from vaccine-preventable diseases.

DTP3 coverage by age 12 months is a major indicator of immunization program performance; coverage with other vaccines, including the third dose of polio vaccine and the first dose of measles-containing vaccine is also assessed. Vaccination coverage is calculated as the percentage of persons in a target age group who received a vaccine dose. Administrative coverage is the number of vaccine doses administered to those in a specified target age group divided by the estimated target population. Countries report administrative coverage annually to WHO and the United Nations Children’s Fund (UNICEF) (2). Vaccination coverage surveys estimate vaccination coverage by visiting a representative sample of households with children in a specified target age group (e.g., 12–23 months). Dates of vaccination are transcribed from the child’s home-based record or are recorded based on caregiver recall. WHO and UNICEF derive national coverage estimates through an annual country-by-country review of all available data, including administrative and survey-based coverage. As new data are incorporated, revisions of past coverage estimates (3,4) and updates are published on their websites (5,6). This report is based on WHO and UNICEF estimates of vaccination coverage.

Estimated global DTP3 coverage among children aged <12 months in 2013 was 84%, ranging from 75% in the WHO African Region to 96% in the Western Pacific and European regions, and representing 111.8 million vaccinated children (Table 1). Approximately 21.8 million eligible children did not complete the 3-dose series; among them, 12.2 million (56%) did not receive the first DTP dose, and 9.6 million (44%) started, but did not complete, the 3-dose series. Estimated global coverage with Bacille Calmette-Guérin vaccine, the third dose of polio vaccine, and the first dose of measles-containing vaccine were 90%, 84%, and 84%, respectively. During 2013, a total of 129 of 194 WHO member states achieved ≥90% national DTP3 coverage, and 56 achieved ≥80% DTP3 coverage in every district. DTP3 coverage was 80%–89% in 31 countries, 70%–79% in 16 countries, and <70% in 18 countries.

Among the 21.8 million children who did not receive 3 DTP doses during the first year of life, 10.9 million (50%) lived in three countries (India [31%], Nigeria [13%] and Pakistan [6%]); 14.8 million (68%) lived in 10 countries (Figure). An estimated 12.2 million (56%) incompletely vaccinated children did not receive the first DTP dose, and nearly 9.6 million (44%) started but did not complete the 3-dose series.

Additional vaccines are increasingly being introduced into national immunization programs. By the end of 2013, hepatitis B vaccine was included in the routine immunization (RI) schedule in 183 (94%) countries; in 93 (58%) countries, a birth dose administered within 24 hours of birth was included to prevent perinatal hepatitis B virus transmission. Worldwide (including countries that have not introduced the vaccine), coverage with 3 doses of hepatitis B vaccine was 81%, and by region ranged from 74% in the South-East Asia Region to 92% in the Western Pacific Region (Table 1). A hepatitis B vaccine birth dose was given to 38% of newborns globally, ranging from 11% in the African Region to 79% in the Western Pacific Region. Rubella vaccine as part of the RI schedule has been introduced in 137 (71%) countries, with an estimated coverage of 44% globally. Coverage with 3 doses of *Haemophilus influenzae* type b vaccine, which had been introduced into 189 (97%) countries* by 2013, was 52% globally, ranging from 18% in the Western Pacific Region to 90% in the Americas Region. By 2013, rotavirus vaccine was introduced in 52 (27%) countries, and pneumococcal conjugate vaccine (PCV) was introduced in 103 (53%) countries. Coverage with the completed rotavirus vaccination series (2 or 3 doses, depending on vaccine used) was 14% globally and reached 70% in the Americas Region. Coverage with 3 doses of PCV was 25% globally and was highest (77%) in the Americas Region. MCV2 was included in the RI schedule in 148 (76%) countries; global coverage in 2013 was 53%.

MCV2 and booster doses for DTP and polio vaccine are administered during the second year of life or later. A total of 159 (82%) countries now have at least one vaccination in the RI schedule during the second year of life. The most common vaccines administered during these visits are MCV2 (57 countries), diphtheria-tetanus (DT)–containing boosters (105 countries), and polio vaccine boosters (78 countries) (Table 2).

## Discussion

Although global coverage estimates for DTP3, Bacille Calmette-Guérin vaccine, the first dose of measles-containing vaccine, and the third dose of polio vaccine have increased substantially since the start of the Expanded Program on Immunization, coverage estimates for these vaccines have plateaued over the past 5 years (7). In 2012, the World Health Assembly endorsed the Global Vaccine Action Plan as a framework for strengthening RI systems. One of the Global Vaccine Action Plan’s guiding principles is to improve equity in access and use of RI services. Nearly 70% of incompletely vaccinated children worldwide live in only 10 countries (50% live in only three countries), highlighting disparities among countries. Two thirds of countries achieved the Global Vaccine Action Plan target of 90% DTP3 coverage nationally, whereas less than one third achieved >80% DTP3 coverage in every district, highlighting coverage disparities within countries.

As immunization systems mature and additional vaccines are incorporated into vaccination schedules, inequity of RI services by age plays a greater role in discrepancies in immunity, and the importance of immunization platforms beyond the first year of life increases. The majority of countries in all WHO regions have incorporated at least one second-year vaccine into the RI schedule, ranging from 51% of countries in the African Region to 97% in the Americas Region (Table 2).

Strengthening the platform for RI services during the second year of life provides several benefits. First, a stronger platform can improve coverage with vaccines scheduled after age 12 months, such as MCV2 and DTP boosters. It also provides a foundation for the introduction of new vaccines anticipated to have scheduled doses during the second year of life, such as malaria vaccine (8). In addition, the second year of life platform provides an opportunity to catch up on vaccines missed during the first year. Findings of a recent modeling study suggest that expanding the age range at which children in Africa are eligible to receive the first dose of measles-containing vaccine could increase coverage substantially (9). Finally, an additional well child visit during the second year of life creates an opportunity to integrate RI services with other health interventions, such as vitamin A supplementation and presumptive treatment for intestinal helminths.

What is already known on this topic?

In 1974, the World Health Organization established the Expanded Program on Immunization to ensure that all children have access to routinely recommended vaccines. Since then, global coverage with the four core vaccines has increased from <5% to ≥84%, and additional vaccines have been added to the recommended schedule. Coverage with the third dose of diphtheria-tetanus-pertussis vaccine by age 12 months is a key indicator of immunization program performance.

What is added by this report?

Estimated global coverage with the third dose of diphtheria-tetanus-pertussis vaccine has remained at 83%–84% since 2009, with estimated 2013 coverage at 84%. Global coverage estimates for the second routine dose of a measles-containing vaccine are reported for the first time in 2013; global coverage was 35% by the end of the second year of life and 53% when including older age groups.

What are the implications for public health practice?

Improvements in equity of access and use of immunization services will help ensure that all children are protected from vaccine-preventable diseases.

Several barriers exist to implementation of a strong platform for vaccination in the second year of life. Implementation of policies to allow RI services beyond the first year of life requires training of health workers and improved vaccine forecasting by immunization program managers to accommodate the need for increased vaccine supply and minimize the likelihood of stock outages. In addition, challenges regarding monitoring need to be considered. For administrative coverage, population size estimates used might be less accurate for older cohorts than for birth cohorts. In coverage surveys, parents of older children are less likely to have home-based vaccination records and are more likely to have poor recall of vaccinations. Health messages can encourage parents to keep home-based records beyond the first year of life.

Enhancing the platform for vaccination in the second year of life should be part of a multifaceted approach to strengthening RI systems. Continued assurance of quality vaccine supply, improved awareness and demand for immunization services by the community, and improvement of delivery services to access hard-to-reach populations and minimize missed opportunities are still critical to improving vaccination coverage, especially in those countries that are home to the majority of incompletely vaccinated children.

## Figures and Tables

**FIGURE:**
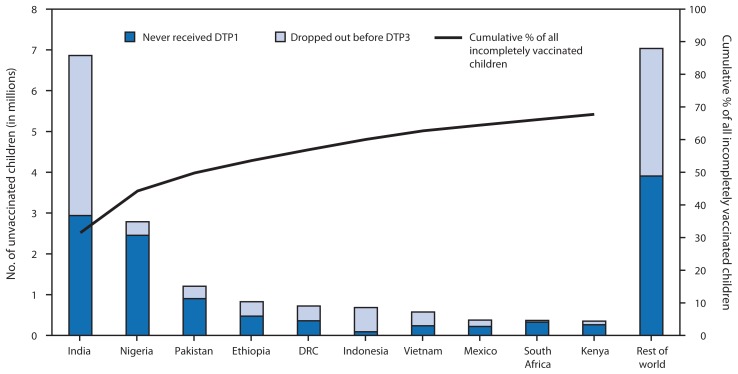
Estimated number of children who did not receive 3 doses of diphtheria-tetanus-pertussis vaccine (DTP3) during the first year of life among 10 countries with the largest number of incompletely vaccinated children and cumulative percentage of all incompletely vaccinated children worldwide accounted for by these 10 countries, 2013 **Abbreviations:** DTP1 = 1 dose of diphtheria-tetanus-pertussis vaccine; DRC = Democratic Republic of the Congo.

**TABLE 1:** Vaccination coverage, by vaccine and World Health Organization (WHO) region* — worldwide, 2013

	Vaccination coverage (%)									
	
WHO region	BCG	DTP3	Polio3	MCV1	MCV2	HepB BD	HepB3	Hib3	Rota last	PCV3
**Total (worldwide)**	**90**	**84**	**84**	**84**	**53**	**38**	**81**	**52**	**14**	**25**
African	83	75	77	74	7	11	76	72	12	35
Americas	94	90	90	92	46	71	89	90	70	77
Eastern Mediterranean	88	82	82	78	65	24	83	60	22	36
European	95	96	96	95	82	41	81	83	3	43
South-East Asia	90	77	76	78	53	26	74	27	0	0
Western Pacific	97	96	97	97	92	79	92	18	4	1

**Abbreviations:** BCG = Bacille Calmette-Guérin; DTP3 = 3 doses of diphtheria-tetanus-pertussis vaccine; Polio3 = 3 doses of poliovirus vaccine; MCV1 = first dose of measles-containing vaccine; MCV2 = second dose of measles-containing vaccine; HepB BD = birth dose of hepatitis B vaccine; HepB3 = 3 doses of hepatitis B vaccine; Hib3 = 3 doses of *Haemophilus influenzae* type b vaccine; Rota last = last dose of rotavirus series; PCV3 = 3 doses of pneumococcal conjugate vaccine.

* Weighted regional average.

**TABLE 2:** Number and percentage of member states with vaccination recommended in immunization schedule during the second year of life, by vaccine and World Health Organization (WHO) region — worldwide, 2013

	No. of member states (%)						
	
WHO region	Total no. of member states	MCV2	DT-containing vaccine	Polio	PCV	Other vaccines	≥1 health care visit during second year
**Total (worldwide)**	**194**	**57 (29)**	**105 (54)**	**78 (40)**	**14 (7)**	**40 (21)**	**159 (82)**
African	47	11 (23)	10 (21)	10 (21)	0	0	24 (51)
Americas	35	4 (11)	31 (89)	28 (80)	3 (9)	11 (31)	34 (97)
Eastern Mediterranean	21	15 (71)	16 (76)	15 (71)	3 (14)	5 (24)	20 (95)
European	53	8 (15)	36 (68)	20 (38)	4 (8)	18 (34)	49 (92)
South-East Asia	11	6 (55)	4 (36)	2 (18)	0	1 (9)	9 (82)
Western Pacific	27	13 (48)	8 (30)	3 (11)	4 (15)	5 (19)	23 (85)

**Abbreviations:** MCV1 = first dose of measles-containing vaccine; MCV2 = second dose of measles-containing vaccine; DT = diphtheria-tetanus; PCV = pneumococcal conjugate vaccine.

## Footnotes

* Includes parts of Belarus, India, and Indonesia.
